# Tibial cortex transverse transport promotes ischemic diabetic foot ulcer healing via enhanced angiogenesis and inflammation modulation in a novel rat model

**DOI:** 10.1186/s40001-024-01752-4

**Published:** 2024-03-06

**Authors:** Wencong Qin, Kaibin Liu, Hongjie Su, Jun Hou, Shenghui Yang, Kaixiang Pan, Sijie Yang, Jie Liu, Peilin Zhou, Zhanming Lin, Puxiang Zhen, Yongjun Mo, Binguang Fan, Zhenghui Li, Xiaocong Kuang, Xinyu Nie, Qikai Hua

**Affiliations:** 1 Department of Bone and Joint Surgery, (Guangxi Diabetic Foot Salvage Engineering Research Center), The First Affiliated Hospital of Guangxi Medical University, Nanning, 530021 Guangxi China; 2 Collaborative Innovation Centre of Regenerative Medicine and Medical Bio-Resource Development and Application Co-constructed by the Province and Ministry, Guangxi Medical University, Nanning, 530021 Guangxi China; 3 Research Centre for Regenerative Medicine, Guangxi Medical University, Nanning, China; 4 National Demonstration Center for Experimental (General Practice) Education, Hubei University of Science and Technology, Xianning, 437100 People’s Republic of China; 5 Yulin Campus of Guangxi Medical University, Yulin, Guangxi China; 6 Department of Neurosurgery, The Third Affiliated Hospital of Zhengzhou University, Zhengzhou University, Zhengzhou, Henan 450052 People’s Republic of China

## Abstract

**Background:**

Tibial Cortex Transverse Transport (TTT) represents an innovative surgical method for treating lower extremity diabetic foot ulcers (DFUs), yet its underlying mechanisms remain elusive. Establishing an animal model that closely mirrors clinical scenarios is both critical and novel for elucidating the mechanisms of TTT.

**Methods:**

We established a diabetic rat model with induced hindlimb ischemia to mimic the clinical manifestation of DFUs. TTT was applied using an external fixator for regulated bone movement. Treatment efficacy was evaluated through wound healing assessments, histological analyses, and immunohistochemical techniques to elucidate biological processes.

**Results:**

The TTT group demonstrated expedited wound healing, improved skin tissue regeneration, and diminished inflammation relative to controls. Marked neovascularization and upregulation of angiogenic factors were observed, with the HIF-1α/SDF-1/CXCR4 pathway and an increase in EPCs being pivotal in these processes. A transition toward anti-inflammatory M2 macrophages indicated TTT's immunomodulatory capacity.

**Conclusion:**

Our innovative rat model effectively demonstrates the therapeutic potential of TTT in treating DFUs. We identified TTT's roles in promoting angiogenesis and modulating the immune system. This paves the way for further in-depth research and potential clinical applications to improve DFU management strategies.

**Supplementary Information:**

The online version contains supplementary material available at 10.1186/s40001-024-01752-4.

## Introduction

Diabetic foot ulcers (DFUs) rank among the most debilitating complications of diabetes mellitus (DM), affecting an estimated 25% of diabetic patients given the global DM prevalence of approximately 6.3% [1]. These ulcers can precipitate a cascade of severe clinical outcomes, including persistent non-healing wounds, infections, osteomyelitis, and limb amputations, culminating in a staggering 5-year post-amputation mortality rate exceeding 70% [2]. The dire implications of DFUs extend beyond the profound impact on the patient's health and quality of life to impose substantial burdens on healthcare systems. Therefore, the quest for efficacious treatments for DFUs is not only a clinical imperative but also a socioeconomic priority.

Diabetic foot lesions, which may involve a range of tissues—including the skin, nerves, blood vessels, muscles, tendons, and bones—necessitate treatment strategies that foster repair and regeneration across these varied structures. Inspired by Ilizarov's pioneering work on distraction osteogenesis (DO)—a process known to induce peripheral tissue regeneration and vascularization [3, 4]—our team has applied a novel technique termed tibial cortex transverse transport (TTT) in the management of severe and intractable DFUs with noteworthy clinical effectiveness. Previous investigations have corroborated that TTT not only augments healing rates in DFUs but also diminishes the rates of amputation and ulcer recurrence, concurrently improving postoperative revascularization of the lower extremities and enhancing foot microcirculation [5–7]. Additionally, our research has revealed that unilateral TTT can facilitate the healing of bilateral DFUs, offering new insights into the treatment of multifocal DFUs [8]. Despite these promising clinical outcomes, the mechanisms underlying TTT's therapeutic impact warrant further exploration.

One likely mechanism by which TTT facilitates healing is through the promotion of angiogenesis in the lower extremities, a process observed in DFU patients following TTT treatment [5–7]. This angiogenic response is thought to be mediated by the activity of endothelial progenitor cells (EPCs) and signaling molecules such as Hypoxia-inducible factor-1α (HIF-1α) and stromal cell-derived factor-1 (SDF-1), as indicated by studies on angiogenesis induced by distraction osteogenesis (DO) [9–11]. In our retrospective clinical study, we observed that TTT can elevate the expression levels of SDF-1 and HIF-1α in patients' peripheral blood, which is consistent with previous research findings [8]. Moreover, TTT is proposed to enhance the healing process by modifying the immune environment, as demonstrated by Yang et al. who reported that TTT induces a shift from M1 to M2 macrophage phenotypes, thus improving local immunity in patients [12]. However, investigating these mechanisms has been historically limited by the lack of an established animal model for TTT. Addressing this gap, our study successfully developed a rat model that closely mimics TTT treatment in humans. This was achieved using a custom external fixator, precise osteotomy techniques, and controlled movement of bone fragments in the rat tibial cortex. Utilizing this model, our controlled experiments allowed us to delve into the effects of TTT on the healing processes of DFUs.

In this study, through comprehensive methods including immunohistochemistry, vascular perfusion studies, and molecular biology, we have delineated the beneficial impacts of TTT in rats. Our findings reveal enhanced angiogenesis and an improved immune environment, establishing a robust platform for future mechanistic studies. These insights significantly advance our understanding of the potential mechanisms by which TTT promotes wound healing in DFUs.

## Materials and methods

### Animals

Specific pathogen-free (SPF) male Sprague–Dawley rats, weighing 350–400 g, were obtained from the Experimental Animal Center of Guangxi Medical University (Guangxi, China). The rats were housed individually under standard feeding conditions. Following the establishment of a diabetic rat model, subjects were stratified into three distinct cohorts based on the intervention received: a Control group, a Sham group, and a TTT group. Each diabetic rat was subjected to full-thickness skin defect surgery followed by HLI to simulate a DFU condition. The TTT group underwent a comprehensive TTT protocol, entailing 10 consecutive days of bone transport. Conversely, the Sham group was subjected to a tibial cortical osteotomy without the application of an external fixation frame or subsequent bone transport. The Control group did not undergo any surgical intervention. Rats presenting with infections or complications at the osteotomy site were omitted from the analysis. Peripheral blood and foot skin biopsies were harvested on postoperative days 10 and 14 for subsequent experimental assays.

### Procedures of an ischemic diabetic foot ulcer model

A 1 week acclimatization period preceded any experimental manipulation. Diabetes mellitus (DM) was chemically induced utilizing a single intraperitoneal injection of streptozotocin (STZ, 55 mg/kg; S8050, Solarbio, China) administered after a 12-h fasting period. Post-induction, blood glucose concentrations were determined from tail vein blood samples at 72-h intervals. Rats demonstrating hyperglycemia, with blood glucose levels persistently at or above 16.7 mmol/l over a span of 10 days, were deemed successfully modeled for DM and included for further study [13].

To faithfully replicate lower extremity ischemia observed in clinical DFU patients, an HLI model was established in diabetic rats. Under deep anesthesia induced by intraperitoneal injection of pentobarbital sodium (50 mg/kg), the right hind limb of each rat was depilated. A precise longitudinal incision was made in the skin overlying the vascular bundle to expose the superficial femoral artery. The artery was then meticulously isolated, securely ligated proximally and distally with a 5–0 suture, and subsequently transected between the ligatures. Following hemostasis, the incision was closed with surgical sutures [14]. Concurrently, a standardized full-thickness skin defect measuring 1 cm × 2 cm was created on the dorsum of the right hind foot using a sterile surgical blade. This dual insult of HLI and cutaneous injury was employed to simulate the complex pathophysiology of DFUs in rats.

### Procedures for TTT in DFU rat model

**Fig. 1:**
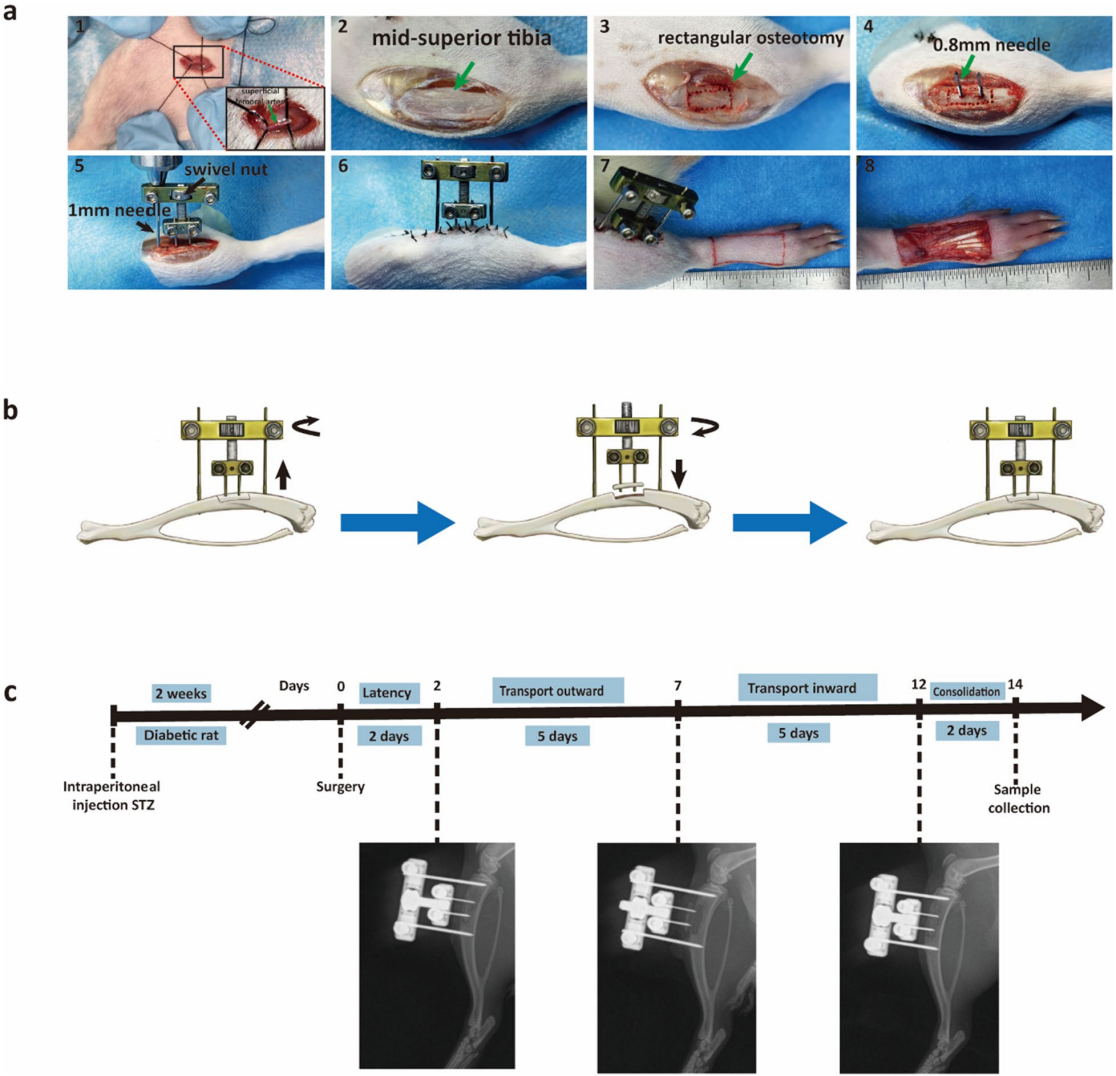
Surgical Procedure of TTT. **a** Creation of an animal model and detailed surgical steps. **b** Illustration of bone transport adjustment using an external fixator. **c** Workflow for the TTT operation and post-operative X-ray imaging is utilized to verify the alignment and status of the cortical bone fragments

In this study, we engineered a bespoke external fixator tailored to the dimensions of the rat tibia. The device comprises four main components: (a) two screws measuring 30 mm in length and 1 mm in diameter for anchoring the frame to the tibial shaft; (b) two miniature screws, 10 mm long and 0.8 mm in diameter, for securing the osteotomized cortical bone segments; (c) adjustable nuts facilitating the mobilization of the cortical fragments; and (d) the frame of the external fixator itself (Fig. 1a).

For surgical implantation, rats were rendered unconscious with an intraperitoneal injection of pentobarbital (50 mg/kg), which was administered to maintain anesthesia throughout the procedure. A meticulous 3-cm longitudinal incision was made inferior to the tibial tuberosity on the right hind limb. Subsequent dissection of the skin and muscle layers exposed the underlying tibia. A rectangular osteotomy, dimensions 4 mm by 8 mm, was fashioned 1 cm distal to the tibial tuberosity using a drill bit with a diameter of 0.6 mm.

At the center of the osteotomized segment, two needles of 0.8 mm diameter were implanted for the purpose of bone transport. An additional pair of needles, each 1 mm in diameter, were inserted proximally and distally to the osteotomy to affix the external fixator to the tibia securely. Care was taken to detach the cortical bone fragment using ophthalmic scissors gently. Once liberated, the screws on the external fixator were tightened to stabilize the apparatus. The mobility of the bone fragment was ensured by adjusting the nuts on the device. The surgical site was then closed with 4–0 silk sutures (Fig. 1a).

### Wound healing rate analysis

Sequential photographic documentation was utilized to monitor the progression of cutaneous wound healing in rats. Digital images were captured immediately following the creation of the skin defect (day 0), and subsequently on postoperative days 3, 7, 10, and 14. Each photograph was timestamped and included a measurement scale for reference. The wound area was quantified using ImageJ software (National Institutes of Health, USA). The percentage of wound healing was calculated using the formula:

$${\text{Wound healing rate }}\left( \% \right)\, = \,[\left( {{\text{Wound area on Day }}0 - {\text{Wound area on Day}}N} \right)/({\text{Wound area on Day }}0\,)]\, \times \,\,{1}00\%$$

### Histology and immunohistochemistry staining

Wound newly formed skin was collected on the 14th day post-surgery and fixed overnight at 4 °C in 4% paraformaldehyde. Following this, tissue sections were prepared using standard procedures. Hematoxylin and eosin staining (HE) was performed on some sections, while Weigert iron hematoxylin and Lixin Red staining were used for Masson staining on other sections. Immunohistochemistry was conducted using the following primary antibodies: CD31 (1:500,GB113151, Servicebio), α-SMA (1:500,GB13044, Servicebio), iNOS (1:1000, No. 22226-1-AP, Proteintech), and CD206 (1:1000, No. 18704-1-AP, Proteintech).

### Blood vessel perfusion

Anesthesia was induced in rats using pentobarbital (50 mg/kg), followed by a precise thoracotomy. A perfusion needle was strategically positioned in the left ventricle, with a simultaneous incision in the right atrium to establish an outflow route for perfusion fluids. The vascular system was first perfused with 100 ml of heparinized saline to prevent coagulation, succeeded by 50 ml of 4% paraformaldehyde for vascular fixation. This was followed by an infusion of 20 ml of MICROFIL^®^ RMV-122 (MV-122, Flow Tech, USA) to enhance vasculature visibility. For complete coagulation of the contrast medium, the rats were refrigerated at 4 °C overnight. Subsequently, hind limb specimens from the operated side were excised for micro-CT scanning. Vessel volumes were quantified and recorded for further analysis.

### Enzyme-linked immunosorbent assay (ELISA)

Peripheral blood was collected from each experimental cohort on the tenth day post-surgery. Serum was subsequently separated for the evaluation of cytokine profiles. Quantitative assessments of hypoxia-inducible factor 1-alpha (HIF-1α, ml059122, mlbio, China), stromal cell-derived factor 1 (SDF-1, ml003227, mlbio, China), vascular endothelial growth factor (VEGF, ml064294, mlbio, China), angiopoietin-1 (ANG-1, ml002916, mlbio, China), angiopoietin-2 (ANG-2, ml002919, mlbio, China), interleukin-10 (IL-10, ml037371, mlbio, China), interleukin-1 beta (IL-1β, ml037361 mlbio, China), and tumor necrosis factor-alpha (TNF-α, ml002859, mlbio, China) were conducted employing ELISA kits.The optical density (OD) of each assay was measured at 450 nm using a microplate reader. Standard curves were generated and the concentrations of cytokines in the samples were calculated according to the manufacturer’s protocols provided with the ELISA kits.

### Flow cytometry

Flow cytometry was employed to quantify the EPCs in the peripheral blood of the rat cohorts. EPCs were identified using a panel of markers: vascular endothelial growth factor receptor 2 (VEGFR2), CD34, and CD133. On the tenth day postoperatively, peripheral blood was drawn and peripheral blood mononuclear cells (PBMCs) were isolated via density gradient centrifugation.The isolated PBMCs were then labeled with Alexa Fluor 647-conjugated anti-VEGFR2 antibody (NB100-530AF647, Novus, USA), fluorescein isothiocyanate (FITC)-conjugated anti-CD34 (SC-7342, Santa Cruz Biotechnology, USA), and phycoerythrin (PE)-conjugated anti-CD133 antibodies (SC-365537, Santa Cruz Biotechnology, USA). Staining was conducted at room temperature shielded from light for 30 min. Post-incubation, the cells were analyzed with flow cytometry to determine the prevalence of EPCs.

### Real time quantitative polymerase chain reaction (RT-qPCR)

Newly formed skin tissues from rat foot were collected on day 10 post-surgery. Total RNA was extracted using RNA extraction kit (DP451, Tiangen, China), and RNA concentration was measured using Nanodrop spe ctrophotometer (NanoDrop Technologies, USA). The RNA was then reverse transcribed using TB Green qPCR Mix (No. RR420A, Takara, Japan), producing cDNA for RT-qPCR. The qPCR primers provided by Sangon Biotech (Shanghai, China) are listed in (Additional file 1: Table S1). The real time PCR conditions were set at 95 °C for 3 min, followed by 35 cycles of 95 °C for 20 s, and 55 °C for 20 s. Experiments were repeated at least three times. β-actin served as the internal reference gene. Gene expression levels were determined using the Ct-method (2^-ΔΔCT^).

### Western blotting

Newly formed skin tissue from the rat foot was collected on the day 10 post-surgery, minced and homogenized in lysis buffer containing a protease inhibitor. After a 30-min incubation period, the mixture was centrifuged to separate the supernatant for total protein extraction. Protein samples (30 μg of total protein) underwent electrophoresis on SDS-PAGE gels and were then transferred to PVDF membranes. The membranes were blocked for 1 h at room temperature and subsequently incubated with primary antibodies overnight at 4 °C. The following day, after incubation with secondary antibody for 1 h, protein bands were visualized using ECL Western Blotting detection reagent (BL520A, Biosharp, China). The primary antibodies used in this study included HIF-1α (1:1000, ab179483, Abcam,USA), SDF-1 (1:1000, 17402-1-AP Proteintech, China), CXCR4 (1:1000, 17402-1-AP, Proteintech, China), CD206 (1:1000, 18704-1-AP, Proteintech, China), iNOS (1:1000, 22226-1-AP, Proteintech, China), COX2 (1:1000, 66351-1-Ig, Proteintech, China) and Arg1 (1:1000, 16001-1-AP, Proteintech, China).

### Statistical analysis

All data were collected from repeated experiments and presented as means ± SEM. To perform the statistical test, GraphPad Prism version 8.0 was used. The differences between experimental groups were analyzed with one-way ANOVA, while multiple comparisons were performed with the least significant difference(LSD) method, with a *P*-value threshold of  < 0.05 set for statistical significance across all analyses.

## Results

### Establishment of TTT in a rat model of diabetic foot

Cortical bone fragment transport was initiated by turning the swiveling nut on the external fixation frame. Following a 2-day postoperative latency period to allow for initial stability, a regimen of precise lateral outward movements of the bone fragments was commenced. Specifically, 1 mm of adjustment per day, in two sessions, for 5 days. Subsequently, after this 5-day outward adjustment phase, the bone fragments were then moved inward at the same rate for an additional 5 days. The entire duration of the TTT procedure, from the beginning of the outward movement to the completion of the inward movement, was 10 days (Fig. 1b). To monitor and confirm the progress of the treatment, postoperative X-rays were taken on days 0, 7, and 12, ensuring that the cortical bone fragments had moved as intended and eventually returned to their original position in the tibia (Fig. 1c).

### TTT enhances wound healing and skin tissue quality in DFU rats

**Fig. 2:**
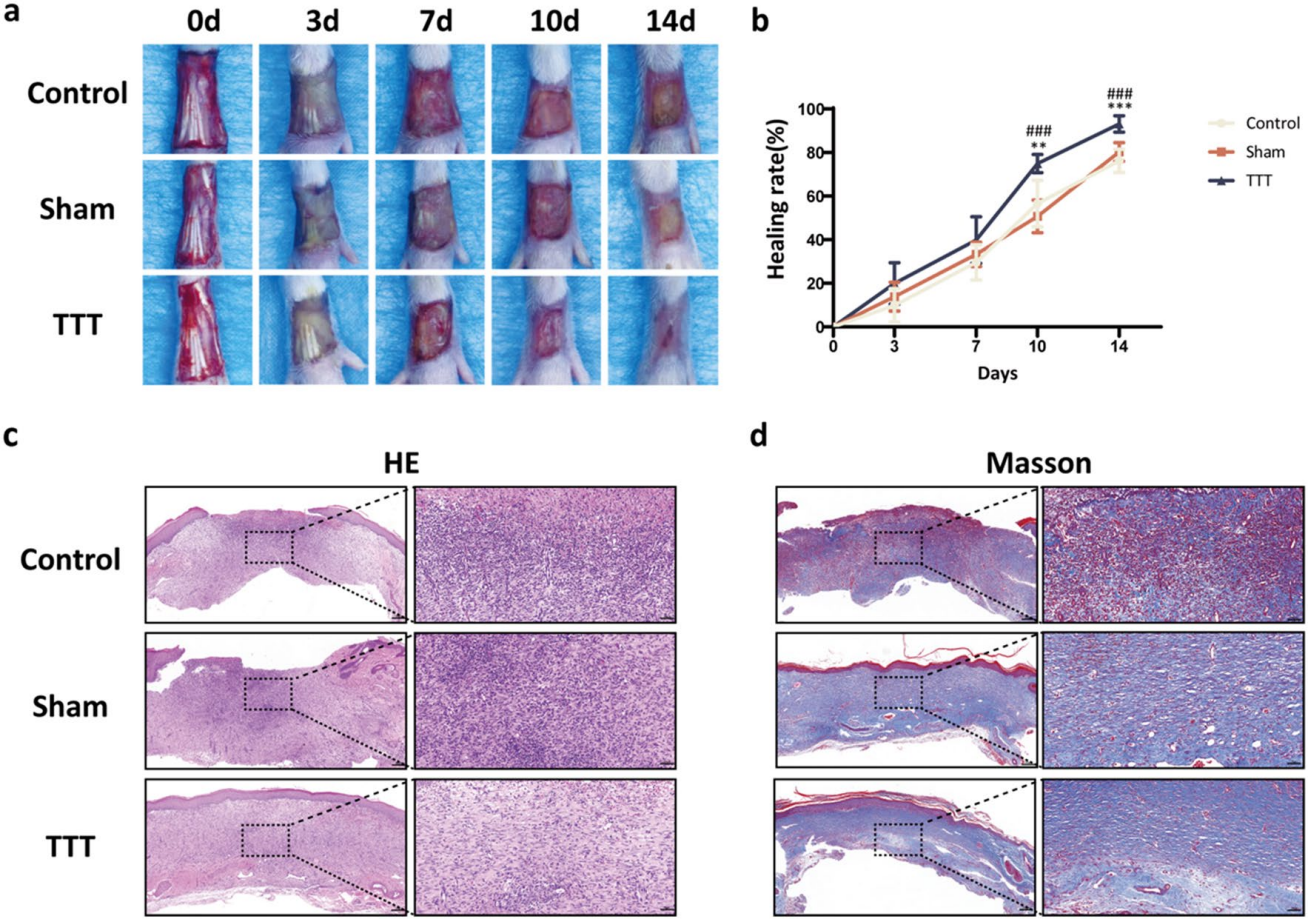
TTT promotes diabetic wound healing and improves the quality of newly generated skin tissue. **a** Representative images displaying wound healing progression and **b** respective wound healing rates across the three groups on days 0, 3, 7, 10 and 14 days post-surgery (*n* = 10). **c-d** H&E and Masson trichrome staining of skin sections from rat feet on day 14 post-surgery. Scale bar = 200 μm (left) and 50 μm (right). *P < 0.05, **P < 0.01, ***P < 0.001, TTT vs. Control; ^#^*P* < 0.05, ^##^*P* < 0.01, ^###^*P* < 0.001, TTT vs. Sham

Quantitative assessment of the wound area using ImageJ software revealed a significant contraction in the TTT-treated group between days 10 and 14 post-surgery, relative to Control and Sham groups. Wound healing rates, calculated as the percent reduction in wound size from day 0 to subsequent days, were markedly higher in the TTT group on days 10 and 14 (*P* < 0.01 for TTT vs Control, *P* < 0.001 for TTT vs Sham on day 10; *P* < 0.001 for both comparisons on day 14). The Control and Sham groups did not differ significantly in healing rates (Fig. 2b).

Histological analysis on day 14 post-surgery showed a well-formed, continuous epithelium in the TTT group, contrasting with the incomplete and varied epidermis in the Control and Sham groups. Inflammation was notably diminished in the TTT group, as evidenced by reduced inflammatory cell presence (Fig. 2c). Furthermore, Masson's trichrome staining indicated a substantial increase in collagen deposition within the TTT group, in comparison to the sparse and disorganized collagen fibers observed in the Control and Sham groups (Fig. 2d).

### TTT enhances angiogenesis in diabetic rats

**Fig. 3:**
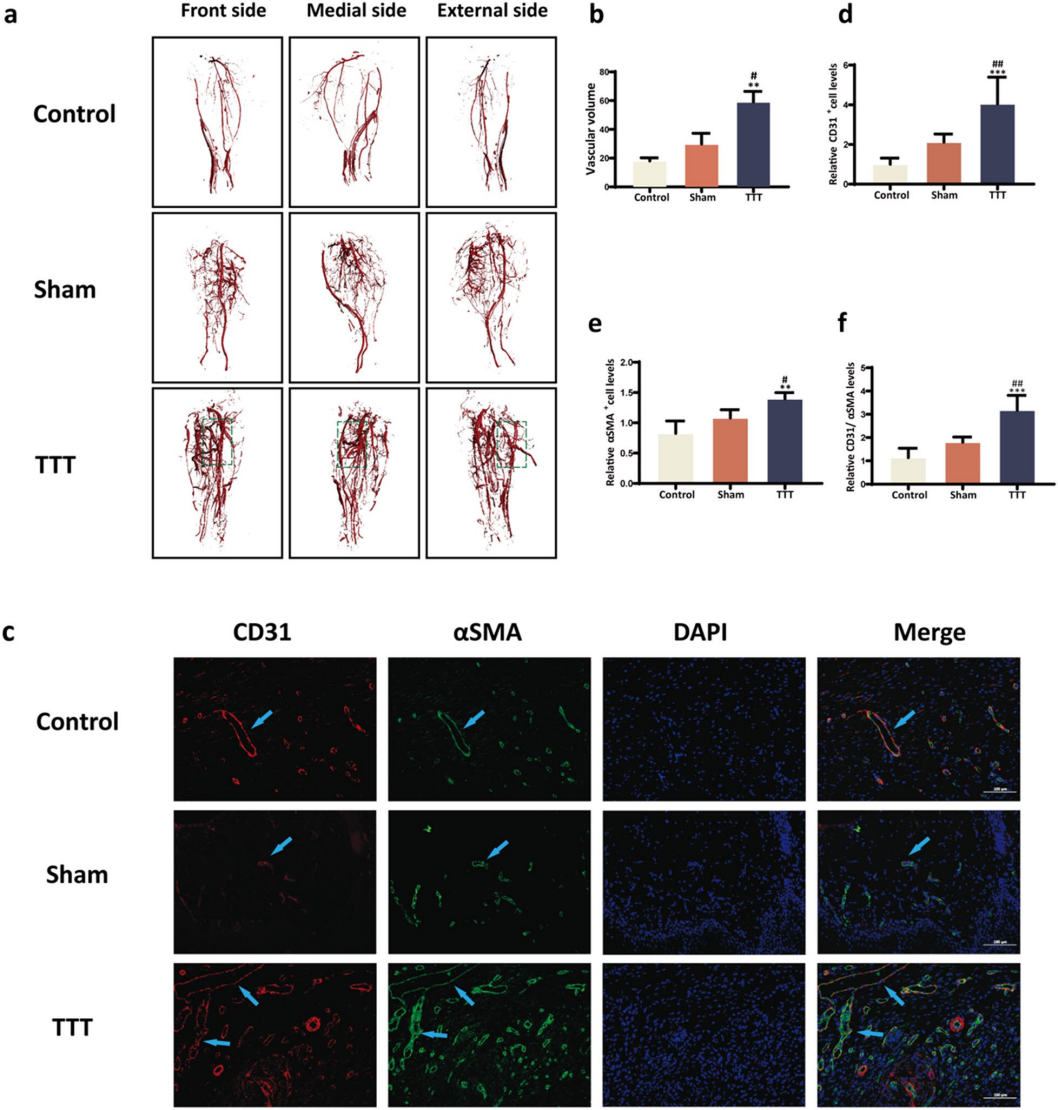
Promotion of Angiogenesis in Diabetic Rats' Hindlimbs and foot ulcers by TTT. **a-b** Representative images alongside quantitative vessel volume analyses from micro-CT scans, post vascular perfusion, in the three study groups (*n* = 4). **c** Dual immunofluorescence staining of wound sections highlighting CD31 (red) and αSMA (green), with nuclear DAPI (blue). Scale bar represents 100 μm. **d-f** Semi-quantitative assessment of CD31, αSMA, and combined CD31/αSMA expressions reveals enhanced angiogenesis in the TTT-treated wounds. Comparative data are normalized to the control group (*n* = 6). Statistical significance indicated as *P < 0.05, **P < 0.01, ***P < 0.001 for TTT versus control; ^#^*P* < 0.05, ^##^*P* < 0.01, ^###^*P* < 0.001 for TTT versus sham

Angiographic analysis on day 14 post-surgery revealed augmented hindlimb revascularization in the TTT group, indicative of superior neovascularization (Fig. 3a). Quantitative evaluations demonstrated a significantly increased vessel volume in the TTT group versus both Control and Sham groups (*P* < 0.01 and *P* < 0.05, respectively; Fig. 3b). Immunofluorescent labeling of CD31 and α-SMA provided additional evidence of enhanced angiogenesis in the TTT group's regenerated skin, highlighted by an increased vessel count within the wound area (*P* < 0.001 against Control, *P* < 0.01 against Sham; Fig. 3c–f).

### TTT augments pro-angiogenic factor expression, EPC mobilization, and activation of the HIF-1α/SDF-1/CXCR4 pathway

**Fig. 4:**
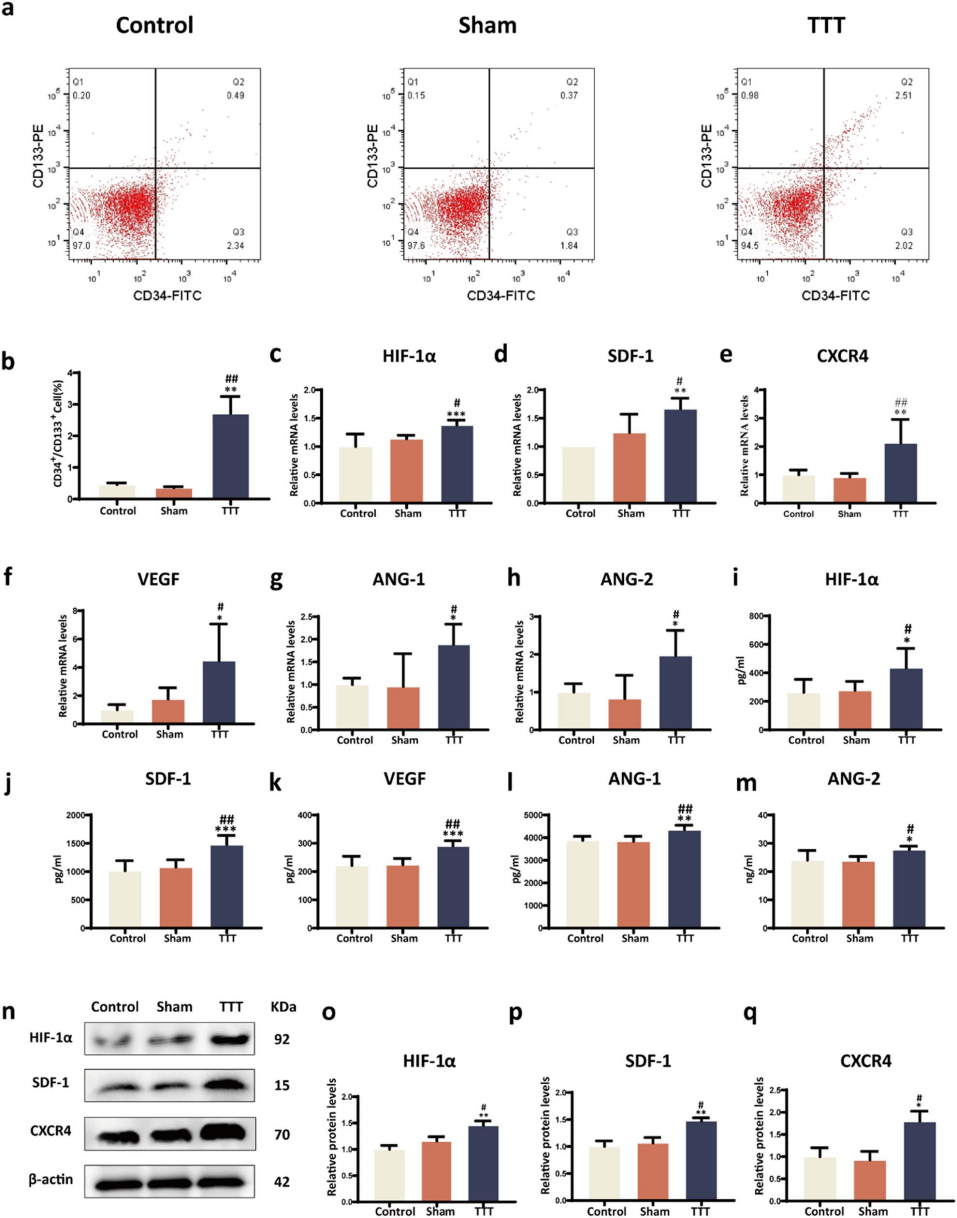
TTT activates the HIF-1α/SDF-1/CXCR4 pathway and stimulates epcs mobilization in diabetic rats. **a** Flow cytometry analysis of VEGR2, CD133, CD34-labeled EPCs in the peripheral blood of diabetic rats (n = 4). **b** Proportion of CD34+/CD133+cells. **c–h** RT-qPCR analysis of mRNA expression levels for genes associated with angiogenesis in wound tissues, including HIF1α, SDF-1, CXCR4, VEGF, ANG-1, and ANG-2 (*n* = 6). **i–m** ELISA results showing the levels of HIF-1α, SDF-1, VEGF, ANG-1, and ANG-2 in the serum of diabetic rats (n = 6).**n–q** Western blot analysis and quantification of HIF-1α, SDF-1, and CXCR4 protein expression in wound tissues (*n* = 6). All protein levels were standardized against β-actin and then normalized to the Control group. *P < 0.05, **P < 0.01, ***P < 0.001 for TTT vs. Control; ^#^*P* < 0.05, ^##^*P* < 0.01, ^###^*P* < 0.001 for TTT vs. Sham

The efficacy of TTT in mobilizing EPCs was assessed via flow cytometry, marking cells with VEGFR2, CD34, and CD133. A considerable increase in circulating EPCs was observed in the TTT group versus the Control and Sham groups (*P* < 0.01, Fig. 4a–b), highlighting TTT's role in enhancing EPCs mobilization, essential for neovascularization. Investigating the underlying molecular mechanisms, the HIF-1α/SDF-1/CXCR4 signaling pathway was analyzed through RT-qPCR and Western blot assays. The pathway's components were significantly upregulated in the TTT group, suggesting an enhanced mobilization and homing capacity of EPCs facilitated by TTT (*P* < 0.05, Fig. 4c–h). ELISA assays conducted on day 10 post-surgery detected significantly higher serum levels of pro-angiogenic factors—HIF-1α, SDF-1, VEGF, ANG-1, and ANG-2—in the TTT group compared to Control and Sham groups (*P* < 0.05, Fig. 4i–m). No significant differences were observed between the Control and Sham groups (*P* > 0.05, Fig. 4i–m). These findings were corroborated by RT-qPCR, which indicated a significant upregulation in the gene expression of VEGF, ANG-1, and ANG-2 in the TTT group (*P* < 0.05, Fig. 4c–h). In further research, Western blotting experiments showed that the HIF-1α/SDF-1/CXCR4 signaling pathway was reactivated. Significant differences were observed between the Control and Sham groups (*P* < 0.05, Fig. 4n–q).

### TTT modulates macrophage polarization and inflammatory mediators

**Fig. 5:**
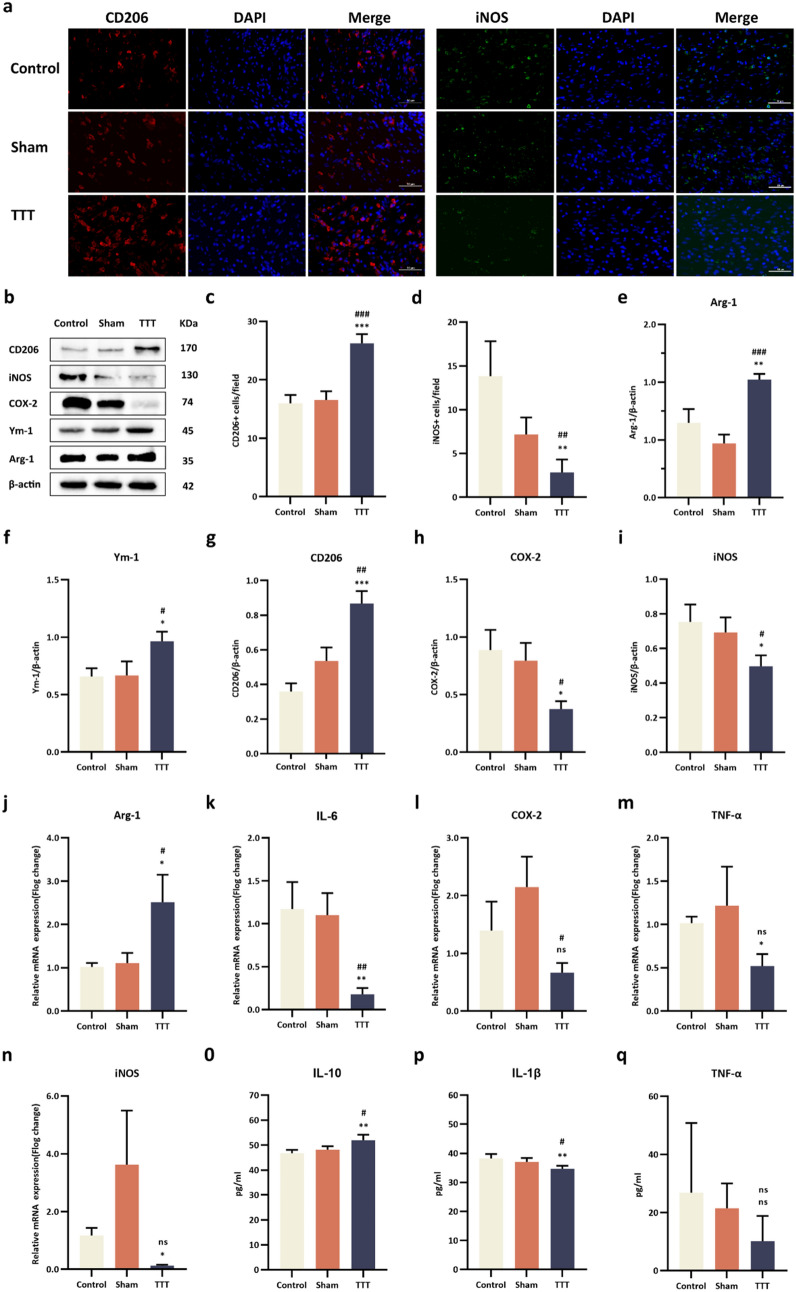
TTT enhances M2 macrophage polarization and modulates inflammatory response in diabetic foot ulcers. **a** Immunofluorescence Analysis: Wound sections were probed with CD206 (red) and iNOS (green) antibodies, and cell nuclei were counterstained with DAPI (blue). Semi-quantitative analysis indicated a marked reduction in M1 macrophages and an augmentation in M2 macrophages within the TTT-treated group. **b–i** Western Blotting and Quantitative Analysis: Investigations were conducted on the expression levels of proteins CD206, iNOS, COX2, Ym1, and Arg1 in wound tissues (sample size: *n* = 6). Protein quantifications were normalized against β-actin. **j–n** Quantitative RT-PCR: mRNA levels of cytokines and enzymes, including IL-6, TNF-α, iNOS, COX2, and Arg-1, were quantified in wound tissues (*n* = 6). **o–q** ELISA Measurements: Concentrations of cytokines IL-10, IL-1β, and TNF-α were measured in the serum of diabetic rats (*n* = 6). Statistical significance is denoted as follows: *P < 0.05, **P < 0.01, ***P < 0.001 for comparisons of TTT versus Control; ^#^*P* < 0.05, ^##^*P* < 0.01, ^###^*P* < 0.001 for TTT versus Sham

**Fig. 6:**
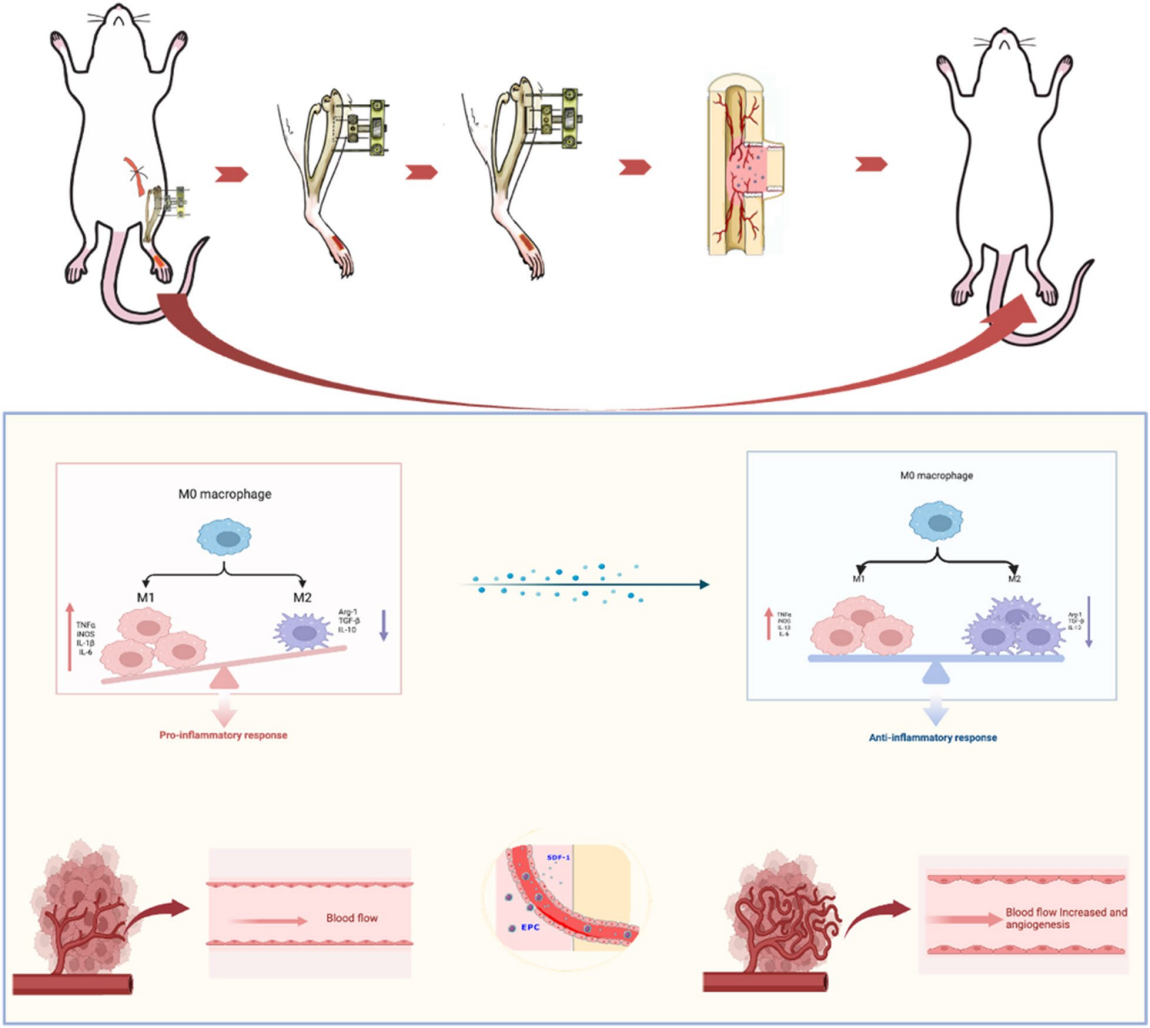
Mechanisms by which TTT promotes healing of ischemic diabetic ulcers. The local tibial cortex of the pulled tibia promotes the expression of angiogenic factors during slow movement and simultaneously regulates the local immune microenvironment of the wound, accelerating wound healing

Macrophage phenotypes in the trabecular area were characterized by iNOS and CD206 immunostaining. On day 14 post-operation, the TTT group displayed a significant reduction in M1 macrophages and an increase in M2 macrophages compared to Control and Sham groups (M1: *p* < 0.05; M2: *p* < 0.001 for both comparisons, Fig. 5a, c, d). Western blot analysis underscored a decline in M1-associated proteins (COX2, iNOS) and a rise in M2 markers (Arg1, Ym1, CD206) in the TTT group on day 10 post-operation (Fig. 5b, e–i). RT-qPCR confirmed these findings at the mRNA level, showing a decrease in pro-inflammatory cytokines (IL-6, TNF-α, COX2, iNOS) and an increase in the anti-inflammatory Arg1 in the TTT group (Fig. 5j–n). Additionally, IL-1β and TNF-α levels were lower, while IL-10 was elevated in the TTT group, highlighting a shift towards an anti-inflammatory profile (Fig. 5o–q, 6).

## Discussion

TTT, an innovative approach for chronic limb ischemic diseases, has shown impressive clinical efficacy in managing diabetic foot ulcers. Retrospective single-center studies, alongside a prospective multicenter cohort study, have validated the effectiveness and safety of TTT for DFUs treatment [6]. Peripheral arterial disease, primarily due to vascular occlusion, is a key factor in the persistence of non-healing diabetic foot ulcers [15, 16]. The robust angiogenic potential of TTT has been demonstrated to significantly enhance lower limb blood circulation. Although Ilizarov’s application of TTT in canine models indicated increased angiogenesis at the localized bone displacement site, the underlying biological mechanisms have yet to be elucidated [4, 17]. A deeper mechanistic understanding necessitated the development of a substantive TTT animal model. Our study introduces such a model in diabetic foot rats, which closely replicates the clinical treatment protocols and their outcomes.

Our findings highlight the therapeutic potential of TTT in a diabetic foot rat model, particularly due to its role in augmenting angiogenesis and alleviating local inflammation at the wound site. These results not only reflect clinical efficacy but also suggest a critical period post-operation—specifically days 10 to 14—where TTT may be optimally effective. This timeframe corresponds with clinical data, suggesting an inherent mechanism of TTT that is particularly active in promoting wound healing during this phase. It implies that a defined duration of bone transport is crucial for TTT to achieve its therapeutic outcomes.

Angiogenesis, critical for enhancing local metabolism, modulating immune responses, and providing nutrients vital for tissue regeneration during wound healing, was conspicuously present in the TTT-treated group [18, 19]. With the aid of CTA vascular reconstruction, we noted a significant increase in angiogenesis at both the hindlimb and bone transport sites in TTT-treated rats compared to control and sham counterparts. The elevated levels of CD31 and αSMA-positive cells in the wound areas further substantiate TTT's capability to stimulate vascular neovascularization, extending from proximal to distal regions of intervention.

Within normal physiological states, the chemokine SDF-1 binds to CXCR4 receptors on EPCs, initiating their mobilization from bone marrow to injury sites to enhance angiogenesis and tissue repair [20, 21]. However, the high-glucose conditions characteristic of diabetes can hinder this process, impairing both the mobilization and angiogenic capacity of EPCs [22, 23]. The therapeutic administration of SDF-1 in diabetic wounds has been reported to counteract this impediment, effectively promoting EPC mobilization and thus improving wound healing outcomes [24]. The critical function of HIF-1α in wound healing processes has been well established; it modulates the activity of its downstream effector, SDF-1, facilitating the recruitment of EPCs to wound sites, which in turn fosters angiogenesis and promotes the healing process [25]. The literature extensively documents the HIF-1α/SDF-1/CXCR4 pathway's integral role in angiogenesis, particularly through the mobilization of stem cells [26, 27]. Distraction osteogenesis is accompanied by significant neovascularization, and research indicates that bone distraction elevates the expression of pro-angiogenic factors like VEGF and SDF-1 in the circulatory system. This increase coincides with an uptick in the number of endothelial progenitor cells, contributing to the observed enhancement in neovascularization. Moreover, the marked upregulation of HIF-1α during distraction osteogenesis, which is associated with bone and angiogenesis, resonates with the findings of our study [28, 29]. Our research identified a rise in the expression of pro-angiogenic factors and an increased presence of peripheral EPCs, alongside the activation of the HIF-1α/SDF-1/CXCR4 pathway in the TTT-treated group. These observations are in harmony with the current body of literature, reinforcing the hypothesis that TTT's efficacy in promoting DFU healing may be largely attributable to its pro-angiogenic influence, which we propose is mediated via the HIF-1α/SDF-1/CXCR4 signaling axis.

Macrophage polarization is a pivotal component in the healing cascade of diabetic wounds [30–33]. The shift from a pro-inflammatory M1 phenotype to a reparative M2 phenotype is essential for transitioning from the inflammatory phase to the healing phase in diabetic wounds. This transition highlights the therapeutic promise of M2 macrophages in the amelioration of diabetic wound healing [34]. Previous studies have demonstrated that TTT facilitates M2 macrophage polarization in non-diabetic models. Our investigation corroborates these findings within a diabetic context, showing that TTT may encourage M2 polarization and mitigate inflammation in diabetic wounds [12]. We observed a significant increase in CD206-positive cells and a decrease in iNOS-positive cells, alongside modifications in macrophage marker proteins, indicating a shift from M1 to M2 macrophage polarization induced by TTT. This macrophage phenotype switch is critical due to the direct correlation between macrophage polarization and inflammation—M1 macrophages are typically associated with pro-inflammatory cytokines, while M2 macrophages are known for their anti-inflammatory and pro-angiogenic outputs [35–37]. Our data, therefore, provide significant insights into the therapeutic mechanisms of TTT. By modulating macrophage polarization, TTT may exert a dual effect on the wound environment: diminishing pro-inflammatory mediators and enhancing anti-inflammatory and pro-angiogenic factor production. This bi-functional modulation suggests that TTT could expedite the healing of diabetic wounds through a synergistic anti-inflammatory and angiogenic response, underscoring its potential as a multifaceted therapeutic strategy.

In our research, we meticulously established a TTT animal model using diabetic rats, demonstrating that TTT can promote the healing of DFUs by regulating angiogenesis and inflammatory pathways. This model, which includes induced hindlimb ischemia in diabetic rats, more faithfully replicates the clinical application of TTT in patients with DFUs and comorbid lower limb ischemia, thus advancing beyond the scope of Yang et al. [12], while our findings are promising, we recognize certain limitations in our study.

## Conclusion

This study not only establishes a novel and effective animal model for studying TTT in DFUs but also sheds light on the mechanisms through which TTT enhances wound healing. We believe that these findings will pave the way for further research and potentially guide the optimized clinical application of TTT for improved patient outcomes in DFUs management.

### Supplementary Information

**Additional file 1: Table S1.** Primers sequences for RT-qPCR.

## Data Availability

The datasets of the current study are available from the corresponding author on reasonable request.
