# Supplementary material for: Tibial cortex transverse transport promotes ischemic diabetic foot ulcer healing via enhanced angiogenesis and inflammation modulation in a novel rat model
Source: Eur J Med Res. 2024 Mar 6;29:155. doi: 10.1186/s40001-024-01752-4 (PMC10918950; doi:10.1186/s40001-024-01752-4)
Supplement: Supplementary file 1 — Additional file 1: Table S1. Primers sequences for RT-qPCR. [file 40001_2024_1752_MOESM1_ESM.docx]

**Tibial Cortex Transverse Transport Promotes Ischemic Diabetic Foot Ulcer Healing via Enhanced Angiogenesis and Inflammation Modulation in a Novel Rat Model**

Wencong Qin^1#^, Kaibin Liu^1#^, Hongjie Su^1,2,3^ , Jun Hou^1,2,3^, Shenghui Yang^1,3^ _,_Kaixiang Pan^1^, Sijie Yang^1,2,3^ ,Jie Liu^1,3^, Peilin Zhou^1^, Zhanming Lin^1^, Puxiang Zhen^1,4^ Yongjun Mo^1^,Binguang Fan^1^, Zhenghui Li^6^ ,Xiaocong Kuang^2,3,5^, Xinyu Nie^1*^  ,Qikai Hua^1,2,3*^

1. Department of Bone and Joint Surgery(Guangxi Diabetic Foot Salvage Engineering Research Center), the First Affiliated Hospital of Guangxi Medical University, Nanning, Guangxi 530021, China.

2. Collaborative Innovation Centre of Regenerative Medicine and Medical Bio-Resource Development and Application Co-constructed by the Province and Ministry, Guangxi Medical University, Nanning, Guangxi 530021, China.

3. Research Centre for Regenerative Medicine, Guangxi Medical University, China.

4. National Demonstration Center for Experimental (General practice) Education, Hubei University of Science and Technology, Xianning 437100, P. R. China

5. Yulin Campus of Guangxi Medical University, Yulin, Guangxi, China.

6. Department of Neurosurgery, The Third Affiliated Hospital of Zhengzhou University, Zhengzhou University, Zhengzhou, Henan 450052, P.R. China.

**Table 1 Primers sequences for RT-qPCR**

| Gene | Forward primer (5′-3′) | Reverse primer (5′-3′) |
| --- | --- | --- |
| VEGF | CACCAAAGCCAGCACATAGGAGAG | CTGCGGATCTTGGACAAACAAATGC |
| ANG-1 | AATACAACACCGTGAGGATGGAAGC | TGTACTGCCTCTGACTGGTTATTGC |
| ANG-2 | CAGTAGCATCAGCCAACCAGGAAG | CCACATGCGTCGAACCACCAG |
| HIF-1α | ACCGCCACCACCACTGATG | GTACCACTGTATGCTGATGCCTTAG |
| SDF-1 | TGAGAGCCATGTCGCCAGAG | ATCCACTTTAATTTCGGGTCAATGC |
| CXCR4 | CAGCCTGTGGATGGTGGTGTTC | GGAGTGTGACAGCTTGGAGATGATG |
| IL6 | ACTTCCAGCCAGTTGCCTTCTTG | TGGTCTGTTGTGGGTGGTATCCTC |
| TNF-α | AAAGGACACCATGAGCACGGAAAG | CGCCACGAGCAGGAATGAGAAG |
| COX2 | CACATTTGATTGACAGCCCACCAAC | AGTCATCAGCCACAGGAGGAAGG |
| iNOS | TCTTGGAGCGAGTTGTGGATTGTTC | AGTGATGTCCAGGAAGTAGGTGAGG |
| Arg1 | GGCGTTGACCTTGTCTTGTTTTGG | GTTCTGTTCGGTTTGCTGTGATGC |
| Thbs4 | CACCAGGACAGCACAGACAAC | TCATCGCATTCATCGCCAATCC |
| β-actin | CGAGGAGTGGTTGCTGAATGAG | GCTTCTTTGCCGTCTGTCCAG |
